# Changes to Gut Microbiota Following Systemic Antibiotic Administration in Infants

**DOI:** 10.3390/antibiotics11040470

**Published:** 2022-03-31

**Authors:** Yoowon Kwon, Young-Sun Cho, Yoo-Mi Lee, Seok-jin Kim, Jaewoong Bae, Su-Jin Jeong

**Affiliations:** 1 Department of Pediatrics, Chungnam National University Sejong Hospital, Sejong 30035, Korea; youyisi68@gmail.com; 2 Department of Pediatrics, CHA Bundang Medical Center, School of Medicine, CHA University, Seongnam 13496, Korea; chocoloys@gmail.com (Y.-S.C.); lymangel@hanmail.net (Y.-M.L.); 3 R&D Institute, BioEleven Co., Ltd., Seoul 06220, Korea; jinkim@bio11.co.kr (S.-j.K.); jbae@bio11.co.kr (J.B.)

**Keywords:** gut microbiome, microbiota, dysbiosis, infant, antibiotic

## Abstract

Long-term antibiotic use can have consequences on systemic diseases, such as obesity, allergy, and depression, implicating the causal role of gut microbiome imbalance. However, the evaluation of the effect of antibiotics in early infancy on alterations to the gut microbiome remains poorly understood. This study aimed to evaluate the gut microbiome state in infancy following systemic antibiotic treatment. Twenty infants under 3 months of age who had received antibiotics for at least 3 days were enrolled, and their fecal samples were collected 4 weeks after antibiotic administration finished. Thirty-four age-matched healthy controls without prior exposure to antibiotics were also assessed. The relative bacterial abundance in feces was obtained via sequencing of 16 S rRNA genes, and alpha and beta diversities were evaluated. At the genus level, the relative abundance of *Escherichia/Shigella* and *Bifidobacterium* increased (*p* = 0.03 and *p* = 0.017, respectively) but that of *Bacteroides* decreased (*p* = 0.02) in the antibiotic treatment group. The microbiome of the antibiotic treatment group exhibited an alpha diversity lower than that of the control group. Thus, systemic antibiotic administration in early infancy affects the gut microbiome composition even after a month has passed; long-term studies are needed to further evaluate this.

## 1. Introduction

The collection of microorganisms, including bacteria, archaea, and eukarya, that colonize the gastrointestinal tract is called the “gut microbiota” [1]. During the host’s life span, the gut microbiota can affect many areas of human health, including modulating innate immunity, maintaining the intestinal mucosal barrier via enhancement of the gut integrity or formation of the intestinal epithelium, protecting against pathogens, providing anti-inflammatory signals to the host, and regulating energy metabolism via essential nutrient synthesis and absorption [1,2,3,4,5,6,7,8]. The construction of the gut microbiota is important as this has the potential to be a major determinant of lifelong health [9]. In infancy in particular, the development of the gut microbiota is highly dynamic and can undergo rapid changes in composition in relation to numerous factors [9] that include the composition of maternal microbiota, mode of delivery, degree of prematurity, administration of perinatal antibiotics, feeding choice (breast versus formula feeding), and environmental elements, such as the presence of pets and siblings [10,11,12,13,14]. A disturbance in gut microbiota composition and function (i.e., gut dysbiosis) can be associated with necrotizing enterocolitis (NEC) in infancy [15], as well as with several chronic diseases in later life [16], including obesity, diabetes, inflammatory bowel disease, cancer, asthma, and allergies, as well as neurological diseases that are associated with the gut–brain axis [17,18]. Several studies suggested that these associations between gut dysbiosis and disease highlight the significance of developing and maintaining a “healthy” gut microbiota [19].

Antibiotics are designed to target and inhibit microorganisms [19] and are widely used to treat various infections to the benefit of public health. However, misuse or overuse of antibiotics can result in antibiotic resistance and short-term effects, including antibiotic-associated diarrhea and gastrointestinal discomfort [19]. Recently, long-term health effects such as microbial imbalance caused by reducing the diversity and perturbing the composition of intestinal microbiota have been suggested [20,21,22]. The application of high-throughput sequencing in both animal and human trials has shown that antibiotics can alter the gut microbiota in particular [19,23,24]. Furthermore, recent studies have tried to evaluate the long-term consequences of antibiotic use on gastrointestinal disease [25], obesity [26,27], metabolic disease [28], and allergic disease [29,30,31]. However, there are few published studies that have evaluated the effect of antibiotics in early infancy on changes in the gut microbiome [19,32]. Therefore, this study aimed to evaluate the effect of systemic antibiotic treatment on the gut microbiome in infants 1 month after delivery and showed that even limited exposure to antibiotics can alter the composition of the infant gut microbiota and decrease its diversity.

## 2. Results

### 2.1. Comparison of Baseline Characteristics between Study Groups

Baseline characteristics, including sex, growth, and delivery mode, did not vary significantly between the antibiotic-treated and control groups (Table 1), and both groups had similar growth patterns according to their weight and height.

### 2.2. Composition of the Gut Microbiota of Antibiotic-Treated and Control Infants

**Figure 1:**
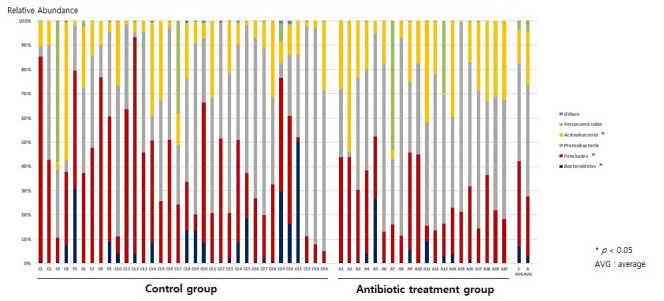
Gut microbiome composition at the phylum level.

We identified bacterial sequences from 54 infant fecal samples (Supplementary Materials). The abundances of the *Firmicutes* and *Bacteroidetes* phyla in feces from the antibiotic treatment group were lower than those of the control group (average relative abundance-antibiotic treatment group (ARA-A) 24.6% vs. average relative abundance-control group (ARA-C) 35.3%, *p* = 0.015, and ARA-A 3.1% vs. ARA-C 7.0%, *p* = 0.049, respectively), while the abundance of *Actinobacteria* was significantly increased to that of the control group (ARA-A 22.4% vs. ARA-C 14.1%, *p* = 0.021) (Figure 1).

**Figure 2:**
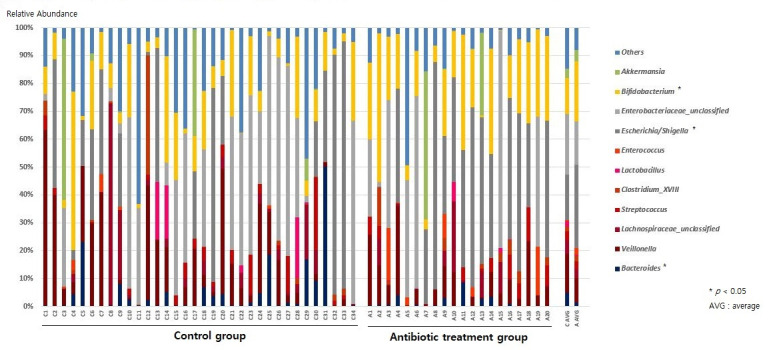
Gut microbiome composition at the genus level.

**Figure 3:**
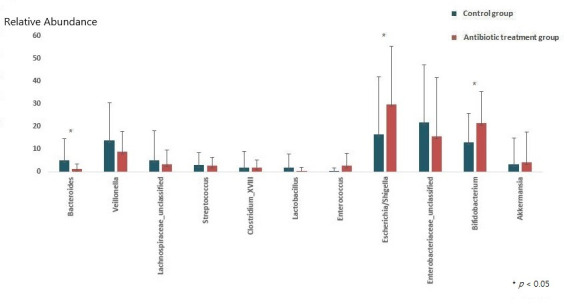
Comparison of gut microbiota between the control and antibiotic treatment groups at the genus level.

At the genus level, the relative abundances of *Escherichia/Shigella* and *Bifidobacterium* increased (ARA-A 29.9% vs. ARA-C 16.4%, *p* = 0.03, and ARA-A 21.4% vs. ARA-C 12.9%, *p* = 0.017, respectively) in the antibiotic treatment group compared with those of the control, while the abundance of *Bacteroides* decreased (ARA-A 1.3% vs. ARA-C 4.9%, *p* = 0.02) (Figure 2 and Figure 3).

**Figure 4:**
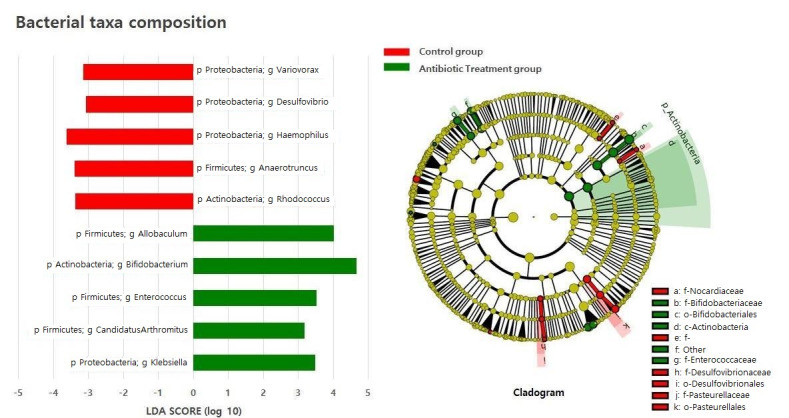
Bacterial taxa composition of the control and antibiotic treatment groups.

We used the linear discriminant analysis (LDA) effect size (LEfSe analysis) to investigate the differences in taxa between groups using the Quantitative Insights Into Microbial Ecology (QIIME) pipeline. The antibiotic treatment group contained *Firmicutes* genera, such as *Allobaculum*, *Enterococcus*, and *Candidatus Arthromitus*, for which their abundances were threefold higher than those in the control group. The abundances of the *Proteobacteria* genera *Klebsiella* and *Actinobacteria* genera *Bifidobacterium* were fourfold and at least threefold higher, respectively, than those in the control group (Figure 4).

### 2.3. Measuring Alpha and Beta Diversity of the Gut Microbiome

**Figure 5:**
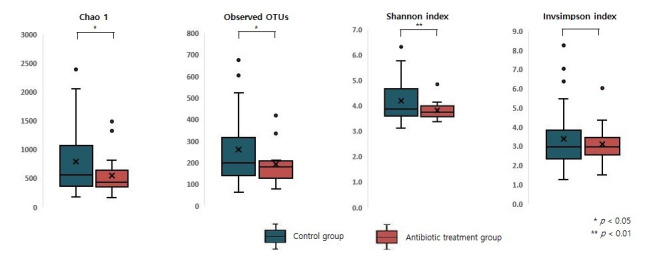
Difference of alpha diversity between the control and antibiotic treatment groups.

Taxonomic analyses suggested that the microbiome of the antibiotic treatment group exhibited an alpha diversity lower than that of the control group (Figure 5). Chao1, observed operational taxonomic units (OTUs), Shannon index, and Invsimpson index were calculated to evaluate the alpha diversity of infant gut microbiome. Chao1 and observed OTUs focus on the richness of diversity; the Shannon index represents diversity evenness and focuses on quantitative and information indices; and the Invsimpson index is a diversity index of dominance indices. There was a significant difference in Chao1, observed OTUs, and Shannon index (*p* = 0.033, *p* = 0.029, and *p* = 0.009, respectively) between the control and antibiotic treatment groups.

**Figure 6:**
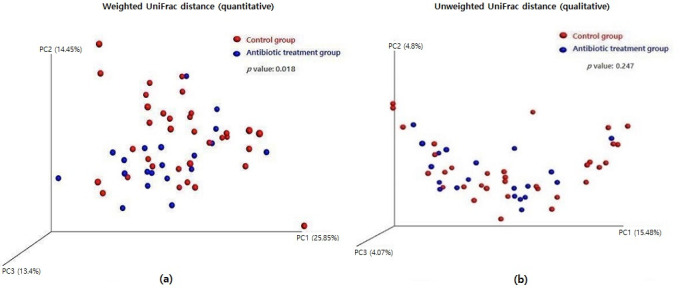
Difference of beta diversity between the control group and the antibiotic treatment group: (**a**) weighted UniFrac distance (quantitative) and (**b**) unweighted UniFrac distance (qualitative).

A three-dimensional principal coordinates analysis (3D PCoA) was performed to visualize the microbial community’s structure based on weighted and unweighted UniFrac distances. A significant change in the microbial community structure with antibiotic treatment was shown in the weighted UniFrac analysis (Figure 6a, *p* = 0.018), but it was not evident in the unweighted UniFrac analysis (Figure 6b, *p* = 0.247).

### 2.4. Difference in Metabolic Activity between Antibiotic-Treated and Control Infants

The Phylogenetic Investigation of Communities by Reconstruction of Unobserved States (PICRUSt) analysis performed demonstrated a significant difference in gut microbiome metabolic activity between the two groups (Table 2). Significantly more genes were expressed in naphthalene degradation, glycolysis gluconeogenesis, and lipoic acid metabolism in the antibiotic treatment group than in those in the control group, whereas this situation was reversed with gene expression in porphyrin and chlorophyll metabolism and fatty acid biosynthesis.

## 3. Discussion

Our study shows that antibiotic administration to infants younger than three months old can impact their gut microbiome. We used 16S ribosomal RNA (16S rRNA) amplicon sequencing to analyze the bacterial community composition found in stool samples obtained from 34 healthy infants and 20 antibiotic-treated infants at least 4 weeks after the end of antibiotic treatment. The gut microbiome differed in composition between both groups, and the lower alpha diversity in the antibiotic treatment group indicated lower microbiome richness and evenness. Moreover, the influence of antibiotics early in life relative to gut microbiota remained even after a few weeks.

Antimicrobial agents are among the most frequently used pharmaceuticals in infants and children. Cox and Blaser recently estimated that by 2 years of age, children in the United States have, on average, received nearly three courses of antibiotics and that this will increase to approximately 10 courses by the age of 10 years [33]. These figures are likely to be relatively similar for other developed countries, although considerable differences in the frequency of antibiotic use have been reported between countries and regions. The use of antibiotics is particularly prevalent in the perinatal period. Intrapartum antibiotic prophylaxis is administered to the mother to prevent preterm birth and to reduce the risk of maternal and neonatal infections. Recent publications indicate that 33–39% of newborn infants are exposed to antibiotics through the mother during delivery [34,35]. In the immediate neonatal period, empirical antibiotic use is also frequent because of the high risk of invasive bacterial infections in the neonate and the difficulty of accurately identifying septicemia in newborn infants. Current guidelines recommend that all symptomatic neonates with a high risk of bacterial infection should receive empirical antibiotic therapy [36]. Consequently, more than 5% of neonates have been reported to receive antibiotics [35] even though the incidence of culture-proven sepsis in newborn infants is less than 0.1% [37].

Previous studies have monitored the intestinal microbiota of infants who underwent antibiotic treatment in the first few days of life and have traced the effects up to 4 and 8 weeks after birth [19,32]. A significantly increased abundance of *Proteobacteria* and decreased abundance of *Bifidobacterium* confirmed that antibiotic exposure at the beginning of life greatly influenced the diversity and composition of infant microbiota. In addition, the microbiota of untreated infants, but whose mothers received antibiotics prior to delivery, showed the same changes observed in the microbiota of treated infants [32,38]. Interestingly, antibiotic exposure, even during pregnancy or lactation, is also associated with the perturbation of infant gut microbiota. Prophylactic antibiotic administration for preventing wound site infection can also affect the different gut microbiome composition of infants born by cesarean section (C-section) compared with that found with vaginal delivery. Vaginally delivered babies traditionally show more *Bifidobacterium* and *Bacteroides* within their gut microbiome [39]; however, in babies born to mothers who received antibiotics prior to delivery, the abundance of *Bacteroides* decreased, corresponding to that observed with babies born by C-section [40]. Similarly, unaffected breast milk mainly contains *Bifidobacterium* [41], but antibiotic treatments during lactation have been shown to lower the abundances of *Lactobacilli* and *Bifidobacteria* and enhance that of mastitis-inducing bacteria in breast milk, causing decreased bacterial diversity [42]. This disruption of the breast milk microbial community and the increased prevalence of mastitis elevated the risk of NEC in the infant because of the decreased intestinal diversity in the first weeks of life [43].

Antibiotic-caused dysbiosis also has an impact on the immune homeostasis due to the disruption of the regulatory T cell (Treg) and T helper (Th) subset balance. The pathophysiology of allergic and autoimmune disease is thought to be driven by the excessive activation of either Th1 or Th2 cells. Additionally, recent studies revealed that Th17 cells and Tregs play a key role in immune homeostasis [44]. The balance between the Tregs and the different Th subsets is vital for immune homeostasis, and antibiotic-induced alteration to the gut microbiota results in the disruption of this immune homeostasis and, finally, to an increase in the risk of asthma and atopic and autoimmune disease [38,45]. Dysbiosis can also affect host immunity and metabolism by altering the production of short-chain fatty acids (SCFAs) production [38]. These metabolites are generated by microbiota in the large intestine, serve as an energy source for colonocytes, and play several roles, including promoting immune and metabolic homeostasis [46]. Physiological concentrations of SCFAs regulate the intestinal barrier function; therefore, the variations in the levels of acetate, propionate, and butyrate can affect the translocation of microbial components and activate the inflammatory response [47]. Furthermore, SCFAs interact with G-protein-coupled receptors to regulate fat deposition [38,48] and are known to play an important role in obesity [49]. Previous studies have suggested a link between antibiotic exposure in infancy and a higher body mass index [50,51] as well as an increased risk of overweight [27,52] and obesity [53] later in childhood [54]. Exposure to antibiotics during the first 6 months of life [50,51,52], or in boys [27,52], has been shown to be more harmful, and long-term antibiotic users (>180 cumulative days of use) had a greater risk of obesity in comparison with short-term users (1 to 30 cumulative days of use) within the first 24 months of age [55]. Thus, an altered gut microbiota can also affect basic immune homeostasis and metabolism with systemic and long-term outcomes in addition to increasing the immediate risk for infection [38]. “Obese gut microbiota” have a lower number of members of the *Bacteroidetes* division and a higher number of members of the *Firmicutes* division. In this study, the bacterial taxa composition of the antibiotics treatment group showed threefold higher abundances of *Firmicutes* than those of the control group (Figure 4).

However, there are conflicting opinions regarding the effects of antibiotic routes of administration on gut microbiota [56]. One study with a porcine model using two beta-lactam antibiotics, oral amoxicillin, and intravenous ertapenem reported that both antibiotics caused significant but distinct alterations in intestinal microbiota composition [57]. Another study suggested that both oral and parenteral antibiotics induced dysbiosis, but oral antibiotics were 100-fold more effective in reducing microbiota colonization [58]. Further research is needed on this issue, but clearly, dysbiosis caused by antibiotics can negatively affect long-term health in a variety of ways [38].

A limitation of this study is that serial the consequences of antibiotics early in life were not documented because of the retrospective nature of the study. The participants were treated with two antibiotics, and the effect of each antibiotic alone was not described. Moreover, all data were represented by relative abundances; no absolute quantification was performed. Therefore, a long-term follow-up evaluation for the participants of our study is needed, and further investigation of the mechanisms of immunology and metabolites should be completed to enable a greater understanding of the gut microbiome.

With the increase in research in microbial communities and the greater availability of “multiomic” techniques [59], many studies have shown that antibiotics also affect gene expression, protein activity, and overall metabolism of the gut microbiota, beyond altering the composition of taxa [38]. Reconstructing the gut microbiota after antibiotic treatment or the use of probiotic bacteria for the purpose of rebuilding the gut microbiota is thought to be an encouraging approach in the future [38].

## 4. Materials and Methods

### 4.1. Study Design and Participants

Twenty infants under 3 months of age who received systemic antibiotic treatments were included in the antibiotic treatment group. They were hospitalized for fever and were treated intravenously with ampicillin/sulbactam 150 mg (as ampicillin dose) per kg per day and cefotaxime 150 mg per kg per day for at least 3 days. All infants in the antibiotic treatment group were born (1) between 37- and 42-weeks gestation; (2) with a birth weight between 2.5 and 4.5 kg; and (3) without any adverse event. Thirty-four age-matched healthy controls who had not been exposed to antibiotics were selected. Stool samples were obtained in both groups. Feces from the antibiotic treatment group were sampled at least 4 weeks following the cessation of antibiotic treatment. We excluded infants who were born (1) from mothers who had group B streptococcus infection and chronic diseases, such as diabetes, hypertensive disorders, or autoimmune disease; (2) from mothers who had taken oral antibiotics during the third trimester of pregnancy; and (3) those born vaginally 12 h after amniotic sac breakage. This study was approved by the Institutional Review Board (IRB) of Ethics Committee of Cha Bundang Medical Center (IRB no. 2017-02-31), and written informed consent was obtained from the parents.

### 4.2. Sample Collection

Fecal samples from newborns were collected from diapers using sterile swabs and were immediately transferred to sterile cryogenic tubes and stored at −20 °C until delivery to the laboratory. Samples were then stored at −80 °C until DNA extraction.

### 4.3. Genomic DNA Extraction

The total genomic DNA was extracted from 200 mg of stool sample using a QIAamp Fast DNA Stool Mini Kit (Qiagen, Germany) with additional bead beating following the manufacturer’s instructions. DNA concentration was measured using a UV-visible spectrophotometer (Nanodrop 2000 c (Thermo Fisher), Waltham, MA, USA). DNA samples were stored at −20 °C for subsequent experimentation.

### 4.4. PCR Amplification of the V3-V4 Region of 16S rRNA Gene

The composition of newborn microbiota was analyzed using 16S rRNA amplicon sequencing with Illumina MiSeq (Illumina, Inc., San Diego, CA, USA). For sequencing, the V3-V4 regions of the bacterial 16S rRNA gene were amplified using primer set F319 (5′-TCGTCGGCAGCGTCAGATGTGTATAAGAGACAGCCTACGGGNGGCWGCAG-3′) and R806 (5′-GTCTCGTGGGCTCGGAGATGTGTATAAGAGACAGGACTACHVGGG TATCTAATCC-3′). DNA templates (12.5 ng/µL) were amplified using a KAPA HiFi Hotstart PCR Kit (Kapa Biosystems, Kenilworth, NJ, USA) with 5 µM of primers. Reaction conditions were as follows: 95 °C for 3 min; 25 cycles of 95 °C for 30 s; 55 °C for 30 s; and 72 °C for 30 s, with a final extension at 72 °C for 5 min. After PCR cleanup, a secondary amplification to attach Illumina Nextera barcodes was performed using i5 forward and i7 reverse primers. The DNA was amplified according to the manufacturer’s protocol. The PCR products were purified using an Agencourt AMpure XP PCR Purification Kit (Beckman Coulter, High Wycombe, UK). The purified products were quantified using a QuantiFluor^®^ ONE dsDNA System (Promega, Madison, WI, USA). The products’ size and quality were evaluated on a Bioanalyzer 2100 (Agilent, Santa Clara, CA, USA). The pooled libraries were sequenced using an Illumina MiSeq instrument with a MiSeq v3 Reagent Kit (Illumina, Inc., San Diego, CA, USA).

### 4.5. Infant Microbial Data Analyses

An analysis of the 16S rRNA gene sequences was performed using the QIIME (v.1.9.1) bioinformatics pipeline (Caporaso et al., 2010). Using qualified sequences (paired-end, Phred ≥ Q20), the OTUs were identified based on an open-reference picking method using 97% identity to entries in the Greengenes database (v13_8) (DeSan-tis et al., 2006) using UCLUST (Edgar, 2010). A sample alpha diversity was calculated using the phylogenetic distance and the number of observed OTUs. For beta diversity comparison between groups, weighted/unweighted UniFrac distances were evaluated (Lozupone et al., 2011). Statistical comparisons of groups were performed with the non-parametric multivariate analysis of variance methods using the Adonis function in the vegan R package. PICRUSt, which is a bioinformatics software package designed to predict metagenome functional content from marker gene (e.g., 16S rRNA) surveys and full genomes, was performed for the analysis of differences in gut microbiome metabolic activity between the two groups. The linear discriminant analysis effect size (LefSE) was determined using the LDA log score (cut-off ≥ 3) to identify distinct taxonomic bacterial biomarkers. The comparisons between groups were analyzed using parametric (Student’s *t*-test) or non-parametric tests (Mann–Whitney U test); a *p* value was assessed as significant when <0.05.

### 4.6. Data Accessibility

Raw data files in fastq.gz format were deposited into National Center for Biotechnology Information (NCBI) database and are available at BioProject under accession number PRJNA802976 (https://www.ncbi.nlm.nih.gov/bioproject/PRJNA802976 (accessed on 4 February 2022)) and Sequence Read Archive (SRA) accession numbers: SRR17868041–SRR17868094.

## 5. Conclusions

Here, we demonstrated that exposure to systemic antibiotics in early infancy can change the composition of the gut microbiome even after a single month.

Antibiotics are fundamentally important, and their use has played a pivotal role in improving human health However, we propose that clinicians consider the administration of antibiotics during early infancy more carefully and try to minimize long-term risk with adjunct treatments by understanding the effects of antibiotic-induced disorders. A long-term follow-up in large samples is needed to study the systemic or metabolic effects of antibiotics.

## Figures and Tables

**Table 1:** Baseline characteristics of children in control and antibiotic treatment group.

Characteristics	Control Group (*n* = 34)	Antibiotic Group (*n* = 20)	*p* Value
Sex (*n*, %)			0.44
Male	20 (58.8)	15 (75)	
Female	14 (41.2)	5 (25)	
Age (month)	4.71	4.55	0.13
Weight (kg)	7.2	7.0	0.67
Height (cm)	67	65	0.38
Delivery mode (*n*, %)			0.77
NSVD	19 (56)	12 (60)	
C-section	15 (44)	8 (40)	

The data are presented as numbers (percent) or means. The abbreviations are as follows: NSVD, normal spontaneous vaginal delivery; C-section, cesarean section. The data were analyzed with descriptive statistics and presented as means, standard deviations, and proportions. Comparisons between groups were analyzed using parametric (Student’s *t*-test) or non-parametric tests (Mann–Whitney U test); a *p* value was assessed as significant when <0.05.

**Table 2:** Kyoto Encyclopedia of Genes and Genomes functional profiling.

		Control Group (*n* = 34)	vs.	Antibiotic Group (*n* = 20)
Level 2	Level 3	LDA	*p* Value	LDA
Xenobiotics biodegradation and metabolism	Naphthalene degradation	-	0.026 ^a^	2.44
Carbohydrate metabolism	Glycolysis gluconeogenesis	-	0.048^a^	2.63
Cofactors and vitamin Metabolism	Lipoic acid metabolism Porphyrin and chlorophyll metabolism	- 2.31	0.018 ^a^ 0.036 ^a^	2.28 -
Lipid metabolism	Fatty acid Biosynthesis	3.09	0.011 ^a^	-
Metabolism of cofactors and vitamins	Porphyrin and chlorophyll metabolism	3.09	0.009 ^b^	

^a^ LDA 2.0 (*p* < 0.05); ^b^ LAD 3.0 (*p* < 0.01). LDA; Linear discriminant analysis effect size.

## Data Availability

The raw data presented in this study are openly available at NCBI database, BioProject under accession number PRJNA802976 (https://www.ncbi.nlm.nih.gov/bioproject/PRJNA802976, accessed on 4 February 2022) and Sequence Read Archive (SRA) accession numbers: SRR17868041–SRR17868094.

## Supplementary Materials

The following are available online at https://www.mdpi.com/article/10.3390/antibiotics11040470/s1: Supplementary file: Number of sequences; refraction curves and relative abundance genus level.
